# Are there phylogenetic differences in salivary tannin‐binding proteins between browsers and grazers, and ruminants and hindgut fermenters?

**DOI:** 10.1002/ece3.6698

**Published:** 2020-08-30

**Authors:** David Ward, Melissa H. Schmitt, Adrian M. Shrader

**Affiliations:** ^1^ Department of Biological Sciences Kent State University Kent OH USA; ^2^ South African Environmental Observation Network Ndlovu Node Phalaborwa South Africa; ^3^ Department of Ecology, Evolution, and Marine Biology University of California Santa Barbara Santa Barbara CA USA; ^4^ School of Life Sciences University of KwaZulu‐Natal Scottsville South Africa; ^5^ Mammal Research Institute Department of Zoology and Entomology University of Pretoria Pretoria South Africa

**Keywords:** African megaherbivores, Afrotheria, hindgut fermenters, ruminants, salivary proteins, tannins

## Abstract

While feeding, mammalian browsers (primarily eat woody plants) encounter secondary metabolites such as tannins. Browsers may bind these tannins using salivary proteins, whereas mammalian grazers (primarily eat grasses that generally lack tannins) likely would not. Ruminant browsers rechew their food (ruminate) to increase the effectiveness of digestion, which may make them more effective at binding tannins than nonruminants. Few studies have included a sufficient number of species to consider possible scaling with body mass or phylogenetic effects on salivary proteins. Controlling for phylogeny, we ran inhibition radial diffusion assays of the saliva of 28 species of African herbivores that varied in size, feeding strategy, and digestive system. We could not detect the presence of salivary proline‐rich proteins that bind tannins in any of these species. However, using the inhibition radial diffusion assay, we found considerable abilities to cope with tannins in all species, albeit to varying degrees. We found no differences between browsers and grazers in the effectiveness of their salivary proteins to bind to and precipitate tannins, nor between ruminants and nonruminants, or scaling with body mass. Three species bound all tannins, but their feeding niches included one browser (gray duiker), one mixed feeder (bush pig), and one grazer (red hartebeest). Five closely related species of small ruminant browsers were very effective in binding tannins. Megaherbivores, considered generalists on account of their large body size, were capable of binding tannins. However, the grazing white rhinoceros was almost as effective at binding tannins as the megaherbivore browsers. We conclude, contrary to earlier predictions, that there were no differences in the relative salivary tannin‐binding capability that was related to common ancestry (phylogeny) or to differences in body size.

## Graphical abstract

Mammalian browsers frequently encounter secondary metabolites such as tannins. Browsers may bind these tannins using salivary proteins, while mammalian grazers likely would not. Controlling for phylogeny, we ran inhibition radial diffusion assays of the saliva of 28 species of African herbivores (browsers, grazers, ruminants, and nonruminants) that differed considerably in size. We conclude, contrary to earlier predictions, that there were no differences in the relative salivary tannin‐binding capability of browsers and grazers, nor ruminants versus nonruminants, that were related to common ancestry (phylogeny) or to differences in body size.

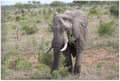

## 1 INTRODUCTION

Large mammalian herbivores foraging in savannas encounter both grasses and woody plants (Searle & Shipley, 2008). Grasses provide lower nutritional value than woody plants because they have a higher dietary fiber content, lower dry matter digestibility, and lower protein content (Demment & Van Soest, 1985; Owen‐Smith, 1982). Woody plants tend to invest less in cell walls but have relatively higher lignin contents than grasses (Ellis, 1990; Iason, Hodgson, & Barry, 1995; McNaughton, Tarrants, McNaughton, & Davis, 1985; Van Soest, 1994). Woody plants tend to defend themselves with plant secondary metabolites (PSMs) unlike grasses (Cooper & Owen‐Smith, 1985; Hofmann, 1989; Lundberg & Palo, 1993; Orians & Ward, 2010; Rohner & Ward, 1997). As a consequence of these fundamental differences between grasses and woody plants, many large mammalian herbivores are adapted to either be grazers (consume primarily (>70%) grasses and forbs) or browsers (consume primarily (>70%) woody plants) (Clauss, Hume, & Hummel, 2010; Clauss & Lechner‐Doll, 2001; Gordon & Illius, 1994; Hofmann, 1989; Hofmann & Stewart, 1972; Robbins, Hanley, et al., 1987; Robbins, Mole, Hagerman, & Hanley, 1987). Hofmann (1973, 1989) proposed that mammalian grazers evolved larger relative stomach capacity, more subdivision of chambers, and smaller openings to those chambers than browsers to increase the retention time of ingesta to improve fiber digestion capability (Clauss & Lechner‐Doll, 2001). Browsing herbivores, on the other hand, benefit from consumption of higher protein in woody plants than in grasses, but often this protein is chemically defended by plant secondary metabolites (PSMs) (Cooper & Owen‐Smith, 1985; Hofmann, 1989; Lundberg & Palo, 1993; Orians & Ward, 2010; Rohner & Ward, 1997; Shrader, Bell, Bertolli, & Ward, 2012). Tannins, a type of PSM, are known to be an antiherbivory defense in many woody plant species (Rhoades & Cates, 1976) and can have numerous deleterious effects when consumed (Shimada, 2006), although their effects depend on the plant species and on the ability of the animal to cope with tannins (Clausen, Provenza, Burritt, Reichardt, & Bryant, 1990; Mole, 1993; Mole, Butler, & Iason, 1990; Mole, Rogler, Morell, & Butler, 1990). The most notable of the effects of plant tannins on mammalian herbivores is a reduction in the amount of protein available and dry matter digestibility (Robbins, Hanley, et al., 1987; Robbins, Mole, et al., 1987). Grasses, on the other hand, are generally low in tannin content, and hydrolyzable tannins are notably absent from them and many other monocots (Chesselet, Wolfson, & Ellis, 1992; Ellis, Foo, & Porter, 1983; Ellis, 1990).

A further subdivision, independent of browsers and grazers, pertains to the fact that herbivores consume plants that have low protein and energy content in comparison with carnivores that consume animals. Much of a plant contains cell walls that are relatively indigestible (Demment & Van Soest, 1985). Hindgut fermenters (or monogastric herbivores) can break down plant material in their hindgut (cecum and colon) and can obtain energy when food is abundant and of high quality (Duncan, Foose, Gordon, Gakahu, & Lloyd, 1990; Steuer et al., 2013). When food becomes limiting and is of low quality, ruminants (or foregut fermenters; “chew the cud”) have four‐chambered stomachs (rumen, omasum, abomasum, and reticulum) to more efficiently break down cell walls (Van Soest, 1994). A third category, known as pseudoruminants (including hippopotamus *Hippopotamus amphibius*), have foregut fermentation, but have only three sections to the foregut (they are missing the rumen, but have the omasum, abomasum, and reticulum) and are not as efficient as ruminants (Clauss et al., 2003).

An additional trait that affects the foraging ecology of mammalian herbivores is body size (Clauss et al., 2003; Demment & Van Soest, 1985). According to the Jarman–Bell principle (Bell, 1971; Geist, 1974; Jarman, 1974), small‐bodied herbivorous mammals (e.g., dik‐dik *Madoqua kirkii* (Manser & Brotherton, 1995)) must be more selective and eat higher‐quality foods (high energy and protein) to maintain their fitness compared to larger‐bodied herbivores because smaller animals have higher mass‐specific metabolic rates and require more protein and energy per unit body mass than larger animals (Van Soest, 1994). Furthermore, small animals have smaller digestive systems which would constrain their abilities to digest fiber (Demment & Van Soest, 1985).

**Figure 1:**
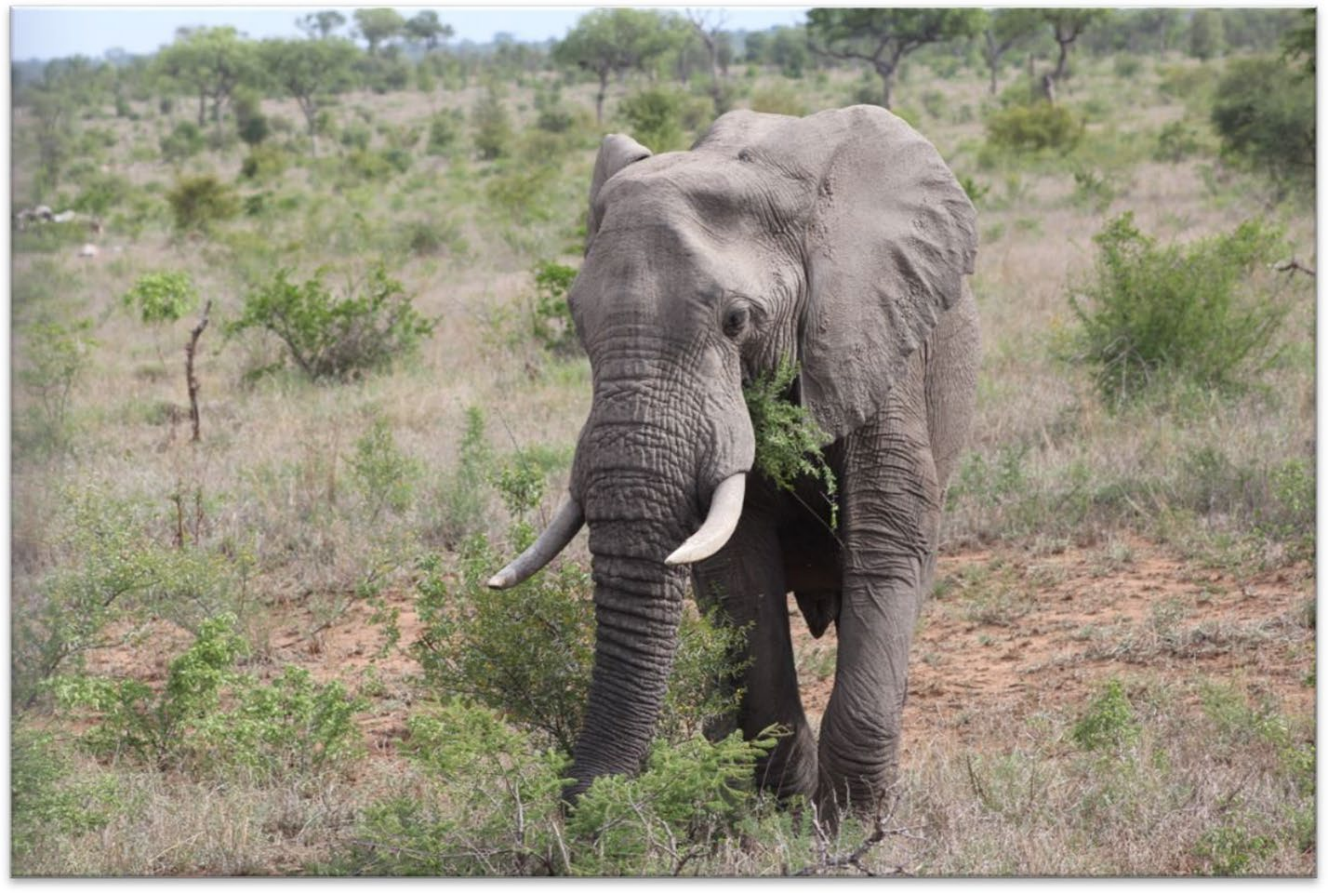
African elephant *Loxodonta africana* browsing on *Vachellia* (formerly *Acacia*) *tortilis*. This species is considered a mixed feeder (consumes both woody plants and grasses). Credit: Megan E. Griffiths

We studied 28 African ungulate species that varied in body size, digestive system, and feeding guild. A number of these African ungulate species are small‐bodied (<15 kg), including red duiker *Cephalophus natalensis*, gray duiker *Sylvicapra grimmia*, blue duiker *Philantomba monticola*, steenbok *Raphicerus campestris*, oribi *Ourebia ourebi*, and klipspringer *Oreotragus oreotragus* (Estes, 1999). We also included five key megaherbivores (weigh >1,000 kg), viz. the black rhinoceros *Diceros bicornis*, white rhinoceros *Ceratotherium simum*, African elephant *Loxodonta africana* (Figure 1), giraffe *Giraffa camelopardalis*, and the hippopotamus (Owen‐Smith, 1988). Many have assumed that large animals can consume large amounts of low‐quality food because they have a low mass‐specific energy requirement and have a large digestive system that can hold a lot of food (Demment & Van Soest, 1985). The Jarman–Bell principle predicts that megaherbivore species should be able to maintain their fitness by being nonselective and eating larger amounts of lower‐quality foods than smaller‐bodied herbivores because larger animals require less energy and protein per unit body mass than smaller animals (Geist, 1974). In the case of the hippopotamus and the white rhinoceros, this would not be difficult because they are grazers and can consume large quantities of food. However, the other species are browsers (black rhinoceros and giraffe) or mixed feeders (also termed intermediate feeders (Hofmann, 1989) African elephant) and consume large quantities of plant biomass to meet their metabolic requirements (Schmitt, Ward, & Shrader, 2016; Shrader et al., 2012). These megaherbivores have high absolute energy requirements, but they also have relatively low mass‐specific energy requirements and have large digestive systems that allow them to consume a lot more forage and effectively digest fiber.

The Jarman–Bell principle takes no account of the role of plant secondary metabolites, such as tannins. However, several authors (Freeland, 1991; Freeland & Janzen, 1974; Westoby, 1978) have argued that mammalian herbivores might be greatly affected by tannins and that they must choose a wide variety of foods to minimize their intake of tannins and sustain their body masses. This may be due to tannin reducing the amount of crude protein available for digestion per bite (Schmitt, 2017; Schmitt, Shuttleworth, Ward, & Shrader, 2018). A strategy for reducing the impact of tannins that mammalian herbivores might employ is the production of salivary tannin‐binding proteins (Hofmann, Streich, Fickel, Hummel, & Clauss, 2008; McArthur, Hagerman, & Robbins, 1991; Shimada, 2006). Tannin‐binding proteins bind to tannins released from foraged material during mastication in the oral cavity (Bennick, 2002; McArthur et al., 1991; Shimada, 2006). These proteins can neutralize some, if not all, of the negative effects of tannins (Harborne, 1993). Salivary tannin‐binding proteins are restricted to mammalian herbivores and omnivores (Harborne, 1993). Mammalian carnivores do not encounter tannins in their diets and do not have salivary proteins. Studies have indicated that browsers have higher levels of salivary proteins than grazers because the former are more likely to encounter tannins (Austin, Suchar, Robbins, & Hagerman, 1989; Hagerman, Robbins, Weerasuriya, Wilson, & McArthur, 1992; Hofmann et al., 2008; Robbins, Hanley, et al., 1987). Proline is a common salivary tannin‐binding protein found in many large mammalian herbivores (Austin et al., 1989; Mole, Butler, et al., 1990; Hagerman 1992). Several browser species have proline in their saliva (mule deer *Odocoileus hemionus* (Austin et al., 1989); moose *Alces* alces (Juntheikki, 1996)), whereas grazers such as sheep *Ovis aries* do not have these salivary proteins (Austin et al., 1989).

**Figure 2:**
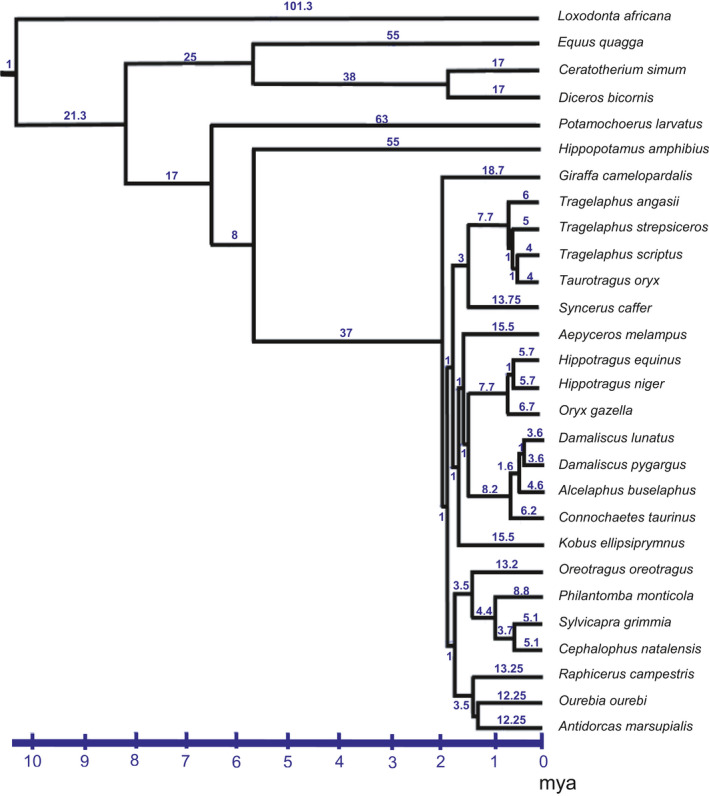
Pruned phylogeny for African ruminants and hindgut fermenters to include only those species that we studied. We used branch lengths (indicated above the branches) as indicated in the *Materials and Methods*, with the shortest branch lengths being set at 1

Large mammalian herbivores may differ in their investments in salivary tannin‐binding proteins not merely because of ecological differences (e.g., browsers versus grazers), their type of digestive system (ruminants vs. hindgut fermenters), or scaling with body size (Hofmann et al., 2008; Pérez‐Barberìa, Elston, Gordon, & Illius, 2004). A key issue of importance is that there are phylogenetic differences among mammal digestive systems (Clauss et al., 2003, 2010; Hofmann et al., 2008; Pérez‐Barberìa et al., 2004; Pérez‐Barberìa, Pérez‐Fernandez, Robertson, & Alvarez‐Enriquez, 2008). For example, ruminants and nonruminants belong to different parts of the mammal phylogeny (Bärmann, Rössner, & Wörheide, 2013; Gatesy, Yelon, DeSalle, & Vrba, 1992; Georgiadis, Kat, Oketch, & Patton, 1990; Hassanin & Douzery, 1999; Matthee & Davis, 2001; Matthee & Robinson, 1999; Figure 2), which may confound claims of adaptation and shared ancestry. However, there are several statistical requirements that need to be addressed before analyzing data that are nested in a hierarchically structured phylogeny. The reason for this is that the absolute values of the species’ values (= “tips”) may be similar because of a shared common ancestry. Such data cannot be assumed to have been drawn independently from the same distribution (Felsenstein, 1985; Huey, Garland, & Turelli, 2019; Ward, 2000; Ward & Seely, 1996). For this reason, phylogenetically independent contrasts are calculated as the absolute differences (“contrasts”) between the “tip” (actual) values that species have and standardized by the square root of the variance, followed by estimates earlier in the phylogeny by similar means (Felsenstein, 1985; Garland, Harvey, & Ives, 1992; Pérez‐Barberìa et al. 2001, 2004, 2008). We specifically examined the phylogenetically independent contrasts of the effectiveness of salivary tannin‐binding proteins for the phylogeny of African mammals belonging to the Afrotheria (Hedges, 2001; Meredith et al., 2011; Springer et al., 1997; Tabuce, Asher, & Lehmann, 2008).

**Figure 3:**
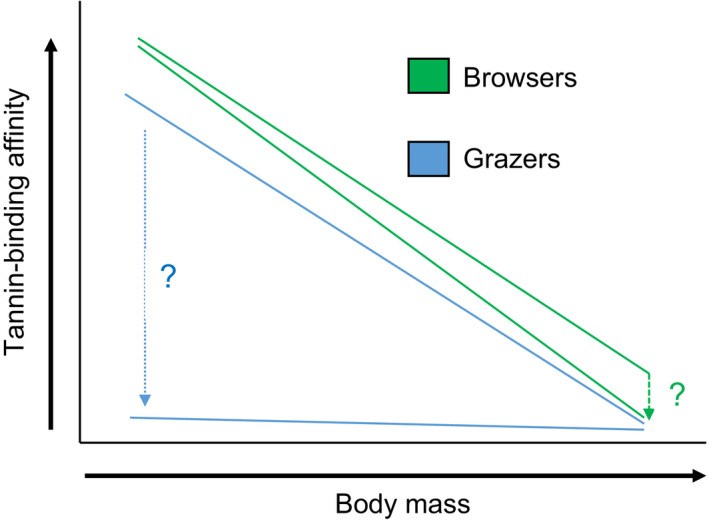
Schematic diagram of predicted differences in salivary tannin‐binding affinity between browsers and grazers relative to body size, assuming that the Jarman–Bell principle works for these salivary proteins too. If grazers do not produce salivary tannin‐binding proteins, they may have uniformly low values regardless of size. If very large herbivores (megaherbivores) are unselective, then tannin‐binding affinity of their saliva should be as low as that of grazers

To explore the physiological response of herbivores to tannins, we tested whether any of the herbivore species have proline‐rich proteins in their saliva. Additionally, we explored the relative binding affinity of herbivore saliva to tannins in an inhibition radial diffusion assay, based on Hagerman's assay (Hagerman, 1987, 2011). We predicted that browsing herbivores may use salivary tannin‐binding proteins as an important mechanism to reduce the negative impact that tannins have on nutritional uptake, and that browsers would invest more in salivary proteins than grazers for the reasons outlined above (Figure 3).

We predicted that:

1. Proline‐rich proteins would be present in the saliva to bind with tannins (Beeley, Khoo, & Lamey, 1991). Browsers, which encounter tannins more frequently in their diets than grazers, would have higher concentrations of proline‐rich proteins than grazers which seldom encounter tannins.
2. Phylogenetic effects would differentiate salivary tannin‐binding proteins of ruminants and nonruminants (hindgut fermenters and pseudoruminants (hippopotamus)) (Demment & Van Soest, 1985) and browsers compared to grazers (Hofmann et al., 2008). Specifically, we predicted that ruminants would be more efficient than nonruminants and pseudoruminants and would have a smaller saliva‐ring size (i.e., bind tannins more effectively) than nonruminants, once the effects of phylogeny had been accounted for. Furthermore, we predicted that browsers, which encounter tannins more frequently than grazers, would have a smaller saliva‐ring size, due to a greater reduction in the effectiveness of tannins (Austin et al., 1989; Hagerman et al., 1992; Hofmann et al., 2008; Robbins, Hanley, et al., 1987; Schmitt et al., 2016; Windels & Hewitt, 2011; Figure 3).
3. Smaller herbivores would invest more in salivary tannin‐binding proteins than larger herbivores because they follow the Jarman–Bell principle (Bell, 1971; Demment & Van Soest, 1985; Jarman, 1974) and require highly digestible energy and protein (Figure 3). Consequently, there should be a significant negative correlation between the saliva‐ring size (i.e., bind tannins more effectively) and the body mass of the species, particularly if they are browsers (Figure 3). Furthermore, if grazers do not produce salivary tannin‐binding proteins, they may have uniformly low values regardless of size (Figure 3). If very large herbivores (megaherbivores) are unselective, then tannin‐binding affinity of their saliva should be as low as that of grazers (Figure 3).

## 2 MATERIALS AND METHODS

### 2.1 Research ethics

All aspects of this study were approved by the University of KwaZulu‐Natal's Animal Ethics Committee (095/13/Animal). Moreover, none of the animals were harmed or put under any additional stress during this study. Due to the noninvasive and voluntary nature of the saliva collection procedure from the human subjects (i.e., the authors and two additional graduate students—see details below), the BioMedical Research Ethics Committee of UKZN indicated that they did not need to review that experimental design or provide ethical approval. We obtained informed consent for the use of human saliva samples in our study. We confirm that all methods were performed in accordance with the relevant guidelines and regulations of the University of KwaZulu‐Natal, South Africa.

### 2.2 Sample collection

We acquired saliva samples from a combination of wild and semitame mammalian herbivores for this study. Saliva samples were collected during the dry season when resources are limited for herbivores in African savannas and when mixed feeders typically shift to feed on more browse (i.e., woody vegetation, which is chemically defended) than grass (i.e., less chemically defended). We would expect that for mixed feeders that shift to use more woody vegetation than grass, there might be the potential for them to have inducible salivary tannin‐binding proteins, which they would likely be using during this time (Ventura‐Cordero, Sandoval‐Castro, Torres‐Acosta, & Capetillo‐Leal, 2017). To collect samples from wild herbivores, we collected saliva from 28 species during planned game‐capture and translocation activities across South Africa. We collected the herbivore samples from wild individuals while they were immobilized by veterinarians during these routine game‐capture procedures. Although sedatives can alter salivary production in mammals (Scully, 2003), we did not observe excessive salivation in any of the individuals from which we collected samples, which has been noted as a common side effect of certain sedatives (Holz, Holz, & Barnett, 1994). The individuals from which we collected samples were captured by several veterinarians who used drug combinations that were mass‐ and species‐specific. Saliva samples were collected as soon as the individual was safe to approach after sedation and while the mouth environment was likely to be as normal as possible.

To collect the saliva, we used cotton swabs and sampled from the entire mouth, each swab touching each part of the oral cavity (tongue, cheeks, and sublingual region). Humans and domestic goats *Capra hircus* were sampled in a similar way. Additionally, to increase sample size, we collected saliva from 6 semitame elephants that forage naturally from Adventures with Elephants near Bela‐Bela, Limpopo Province, South Africa. These elephants were awake and willingly allowed us to take saliva samples. We immediately sealed the swabs in plastic Eppendorf vials and froze the samples until laboratory analysis. Samples were then thawed, and the saliva was separated from the cotton swabs by centrifuging them at 800 rpm (72 g) for 5 min. The herbivore species we collected, their sample sizes (i.e., number of individuals from which we took samples), feeding niches, and gut morphologies are listed in Table 1.

**Table 1:** Large mammalian herbivores sampled for these analyses, indicating their feeding niches, gut morphologies, and their effects on tannin binding (mean ring diameter)

Species	*N*	Feeding Niche	Digestive system	Mean ring diameter ± *SD* (mm)
Black Rhinoceros *Diceros bicornis*	10	Browser	Nonruminant	0.4 ± 0.66
Blesbok *Damaliscus pygargus*	10	Grazer	Ruminant	1.8 ± 1.03
Blue Duiker *Philantomba monticola*	2	Browser	Ruminant	0.4 ± 0.53
Buffalo *Syncerus caffer*	10	Grazer	Ruminant	1.3 ± 0.95
Bush Pig *Potamochoerus larvatus*	2	Mixed feeder	Nonruminant	0.0 ± 0.00
Bushbuck *Tragelaphus sylvaticus*	9	Browser	Ruminant	0.8 ± 1.03
Eland *Taurotragus oryx*	13	Mixed feeder	Ruminant	1.5 ± 1.19
Elephant *Loxodonta africana*	10	Mixed feeder	Nonruminant	1.4 ± 0.46
Gemsbok *Oryx gazella*	4	Browser	Ruminant	3.0 ± 0.41
Giraffe *Giraffa camelopardalis*	10	Browser	Ruminant	0.6 ± 1.26
Gray Duiker *Sylvicapra grimmia*	2	Browser	Ruminant	0.0 ± 0.00
Hippopotamus *Hippopotamus amphibius*	4	Grazer	Foregut fermenter	2.4 ± 0.58
Impala *Aepyceros melampus*	10	Mixed feeder	Ruminant	1.3 ± 0.82
Klipspringer *Oreotragus oreotragus*	4	Browser	Ruminant	1.1 ± 1.31
Kudu *Tragelaphus strepsiceros*	10	Browser	Ruminant	0.1 ± 0.32
Nyala *Tragelaphus angasii*	10	Browser	Ruminant	0.7 ± 0.82
Oribi *Ourebia ourebi*	2	Grazer	Ruminant	1.8 ± 0.35
Red Duiker *Cephalophus natalensis*	10	Browser	Ruminant	0.9 ± 0.91
Hartebeest *Alcelaphus buselaphus*	3	Grazer	Ruminant	0.0 ± 0.00
Roan *Hippotragus equinus*	5	Grazer	Ruminant	0.1 ± 0.22
Sable *Hippotragus niger*	10	Grazer	Ruminant	1.2 ± 0.57
Springbok *Antidorcas marsupialis*	10	Grazer	Ruminant	1.2 ± 0.83
Steenbok *Raphicerus campestris*	4	Browser	Ruminant	2.3 ± 0.65
Tsessebe *Damaliscus lunatus*	10	Grazer	Ruminant	0.6 ± 0.76
Waterbuck *Kobus ellipsiprymnus*	10	Grazer	Ruminant	0.6 ± 0.78
White Rhinoceros *Ceratotherium simum*	4	Grazer	Nonruminant	0.8 ± 0.65
Blue Wildebeest *Connochaetes taurinus*	9	Grazer	Ruminant	1.3 ± 0.80
Burchell's Zebra *Equus quagga*	10	Grazer	Nonruminant	1.4 ± 0.57

Note

A smaller ring means a greater effect on tannins. Nonruminant = hindgut fermenter. Mixed feeders consume both woody plants and grasses, albeit not necessarily in the same place (Codron et al., 2012; Schmitt et al., 2016; Shrader et al., 2012). *N* = sample size (number of individuals). We also sampled five human individuals (positive control) and five domestic goats (negative control) (see Section 2).

### 2.3 Proline‐rich proteins

To test for the presence of proline‐rich proteins in the herbivore saliva, we used two different approaches. First, we used a sodium dodecyl sulfate–polyacrylamide gel electrophoresis (SDS‐PAGE) (Laemmli, 1970). We used a 12.5% running gel and 4% stacking gel to separate the various proteins present in the herbivore saliva. We used a Bradford’s (1976) analysis to assess the amount of protein in each sample. Thereafter, we diluted each saliva sample by 50% with phosphate‐buffered saline (PBS) to reduce the potential negative effects of salts in our gels. We ran reducing gels, so we added β‐mercaptoethanol, a reducing agent that separates proteins into their most basic forms or subunits, in a 1:10 ratio, to the sample buffer prior to addition of the saliva samples (Beeley et al., 1991). We loaded each gel with 25 μg/μl of protein and ran them at 18 mA per gel until the dye front reached 0.5 cm from the edge of the gel. For each gel, we tested the saliva of each of the 28 herbivore species against a negative control (i.e., lacks proline‐rich proteins; domestic goat saliva) and a positive control (i.e., has proline‐rich proteins; human saliva).

The second approach we used was to run a comparative SDS‐PAGE gel first using the staining and destaining method (Laemmli, 1970), as well as the Beeley et al. (1991) method for staining and destaining to probe for proline‐rich proteins. For Laemmli’s (1970) method, we mixed 45% (v/v) methanol, 10% (v/v) acetic acid, and 0.25% (w/v) Coomassie Brilliant Blue R‐250. We left gels to stain overnight. The gels were then destained with 50% (v/v) methanol and 10% (v/v) acetic acid. Gels in this treatment were compared to gels that were stained in 0.1% w/v CBB R‐250 mixed with 40% v/v ethanol and 10% v/v acetic acid for 3 hr and then destained for 4 d in 10% v/v acetic acid (Beeley et al., 1991). Should any of the herbivore samples contain proline‐rich proteins according to the Beeley et al. (1991) technique, they should stain pink/violet. We were unable to take photographs of the gels because the University of KwaZulu‐Natal did not have adequate color photography. Black‐and‐white images do not show the pink bands effectively, and conventional SLR cameras/flash do not photograph such bands successfully.

### 2.4 Relative tannin‐binding capacities

To test the tannin‐binding capabilities of herbivore saliva, we altered Hagerman’s (1987) radial diffusion assay to test for relative tannin–protein precipitation (Hagerman, 2011). We used this assay to understand relative tannin precipitation capabilities by herbivore saliva, not to test for specific salivary tannin‐binding proteins or absolute tannin‐binding levels. This inhibition assay tested for a reduction in the size of the ring relative to pure tannic acid. To make the radial diffusion plates, we followed Hagerman’s (2011) procedure that precipitates tannin using bovine serum albumin (BSA). We made a series of 6 μL deep wells in an Ouchterlony double‐immunodiffusion pattern (Ouchterlony, 1953) in place of the traditional 4 wells per plate pattern (Hagerman, 1987). Instead of performing the classic radial diffusion assay, we created an inhibition assay whereby a tannic acid solution was mixed with saliva prior to being pipetted into the wells. For this experiment, we used a concentration of 1 g tannic acid per 100 ml, which was suspended in 70% acetone. The saliva may bind with tannins in the extract prior to contact with the bovine serum albumin mixed into the agar, thus making it unavailable for binding to the BSA. The resulting saliva tannin precipitate rings in the agar could then be compared to rings formed by the tannic acid stock solution alone as an indication of whether there was a difference between the amounts of tannins bound in each treatment (Bryant et al., 1991; Robbins, Hagerman, Austin, McArthur, & Hanley, 1991). For this experiment, we used a concentration of 1 g tannic acid per 100 ml, which was suspended in 70% acetone (Alonso‐Díaz et al., 2008; Ventura‐Cordero et al., 2017). Hagerman (2011) indicates that acetone‐containing extracts do not inhibit the precipitation reaction like other protein‐precipitating methods, so acetone can be used with this method. Because the aim of this assay was to make comparisons across the herbivore species and their relative abilities to precipitate tannins (and not identify absolute tannin‐binding capabilities), we used a standardized concentration of a common type of tannins for this assay. Ultimately, this provided a comparative measure of the tannin‐binding ability of the saliva.

To test for the tannin‐binding capacities of herbivore saliva, we tested 4 μl of the tannic acid solution alone as well as 4 μl of the tannic acid solution combined with 2 μl of herbivore saliva. Because we only aimed to make comparisons of the tannin‐precipitating abilities of the saliva of different herbivore species and were not making absolute estimates of their tannin‐binding abilities, we used a 2:1 ratio of tannic acid solution to saliva because it allowed us to identify any reaction at the scale of the wells. Prior to the addition of the tannic acid solution and saliva mixture to the wells, we mixed the two components in Eppendorf vials, vortexed them for 5 s, and allowed the mixture to react at room temperature (~25°C) in artificial (laboratory) light for 30 min. After reacting for 30 min, we pipetted the solution into the wells. We sealed each petri dish with parafilm and placed them into an incubator at 30°C for four days (Hagerman, 2011). On day four, we measured the perpendicular diameters of the tannin‐binding ring that had formed around each well and took an average (Hagerman, 2011).

### 2.5 Statistical analyses

We used mean ring diameter (mean of the perpendicular measurements of each tannin‐binding ring) for the tannic acid solution as our dependent factor and treatment (digestive system and feeding niche) as the independent factor. We also tested for an interaction effect between digestive system and feeding niche. We considered the pseudoruminant (hippopotamus—Table 1) as a nonruminant because it is less efficient than a ruminant. To account for normality and homogeneity of variance, we transformed the data, using the reciprocal of the mean saliva‐ring distance, and calculated the log_10_ of body mass. We ran an ANCOVA with reciprocal of the mean saliva‐ring distance as our dependent variable, digestive system and feeding niche as independent variables, and log_10_ body mass as a covariate. We also ran a regression between the reciprocal of the mean saliva‐ring distance and the log_10_ of body mass.

We primarily used a phylogenetically independent contrast (PIC) approach, using the Brownian motion model of evolution that underlies PIC (Garland et al., 1992). We first compiled a phylogeny based on the Tree of Life Web Project (Maddison, Schulz, & Maddison, 2007) and other papers (Bärmann et al., 2013; Buntjer, Otsen, Nijman, Kuiper, & Lenstra, 2002; Gatesy, Amato, Vrba, Schaller, & DeSalle, 1997; Gatesy et al., 1992; Georgiadis et al., 1990; Hedges, 2001; Matthee & Davis, 2001; Matthee & Robinson, 1999; Meredith et al., 2011; Springer et al., 1997; Tabuce et al., 2008) with a focus on the fact that African ungulates (and other African taxa) are now recognized as belonging to the Afrotheria (Figure 2). We excluded species that were not part of the phylogeny. We used fossil ages as calibrations for the phylogeny (Gatesy et al., 1992; Hedges, 2001) and calculated branch lengths (Meredith et al., 2011). We ran the analysis in the modular comparative method program *Mesquite* (Maddison & Maddison, 2018) using PDAP (Garland et al., 1992). Our categorical independent contrast variables were digestive system and feeding niche and the covariate log_10_ body mass.

We also calculated phylogenetic signal (Münkemüller et al., 2012). Phylogenetic signal of continuous traits, such as saliva‐ring diameter contrasts, is an important measure of the statistical dependence among species’ trait values due to their phylogenetic relationships (Revell, Harmon, & Collar, 2008). Münkemüller et al. (2012) reviewed several methods of assessing phylogenetic signal and found that Blomberg, Garland, and Ives (2003) to be one of the most useful under a wide range of phylogenetic models. Blomberg et al. (2003) suggested that a reliable way to assess phylogenetic signal is to examine the ratio of the MS Factor (using phylogenetic contrasts) relative to the MS Error. In this case, we analyzed the saliva‐ring‐independent contrasts versus feeding niche‐independent contrasts (browser vs. grazer vs. mixed feeder). A large value for the MS Factor relative to MS Error indicates a significant phylogenetic signal (Münkemüller et al., 2012).

## 3 RESULTS

### 3.1 Proline‐rich proteins

Using the assay of Beeley et al. (1991), we found that our positive control (human saliva) yielded several pink/violet bands indicating the presence of proline‐rich proteins (Beeley et al., 1991), whereas our negative control (domestic goat saliva—Lamy et al., 2008) had numerous protein bands but lacked proline‐rich proteins indicated by the absence of a pink/violet band(s). However, all of the 28 herbivore species had proteins in their saliva, but none of the herbivore samples contained proline‐rich proteins (i.e., no pink/violet bands).

### 3.2 Phylogenetic differences

In these inhibition radial diffusion assays, an effective salivary tannin‐binding protein would reduce the size of the ring (diameter) relative to tannic acid. In our phylogenetic analyses of 28 African ungulate species, there was no significant difference using independent contrasts in digestive system (ruminant vs. nonruminant) for the reciprocal of saliva‐ring size of these species (*F* = 0.088; *p* = .769). Similarly, we found no significant difference using independent contrasts between feeding niches (browsers vs. grazers) in terms of the reciprocal of saliva‐ring size in these ungulate species (*F* = 1.620; *p* = .214). We found that there was a negative correlation (*r* = −0.33) between the reciprocal of saliva‐ring diameter and (continuous) log_10_ body mass‐independent contrasts. However, this relationship was not significant (*F* = 3.217; *p* = .085). Consequently, we could not look for outliers that may reflect species that differed significantly from the 95% confidence intervals (Garland & Ives, 2000). There was also no significant relationship between mean saliva‐ring diameter and log_10_ body mass (*r* ^2^ = 0.004, *F* = 0.072, *p* = .791) for ruminants only.

There was no significant phylogenetic signal for saliva‐independent contrasts versus feeding niche‐independent contrasts (*F* = 0.007, *p* = .933) according to the technique of Blomberg et al. (2003). Similarly, we found no significant phylogenetic signal for digestive system (*F* = 0.373; *p* = .547). Thus, because we found no phylogenetic signal, we did not perform further phylogenetically appropriate comparative analyses such as phylogenetic generalized least squares (PGLS—Symonds & Blomberg, 2014). We note that Blomberg, Lefevre, Wells, and Waterhouse (2012) recognize that phylogenetically independent contrasts (PICs) and phylogenetic generalized least squares (PGLS) approaches are functionally similar.

### 3.3 Ignoring phylogeny

**Figure 4:**
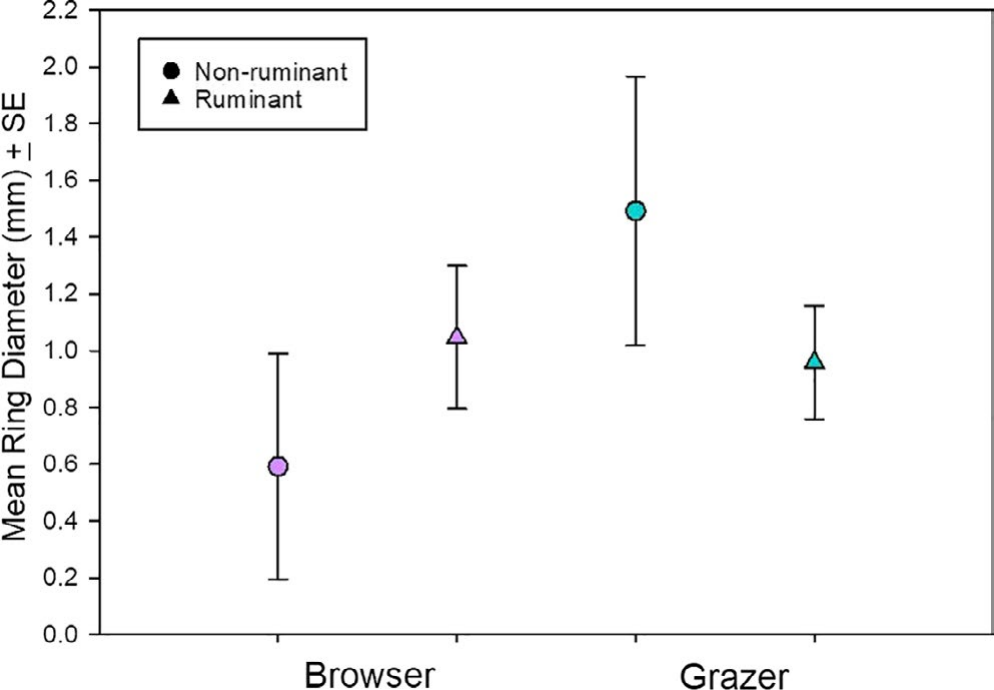
Mean tannin ring diameter ± *SE* for browsers and grazers, differentiated by digestive system. Nonruminants are hindgut fermenters, with the exception of the hippopotamus, which is considered a pseudoruminant

**Figure 5:**
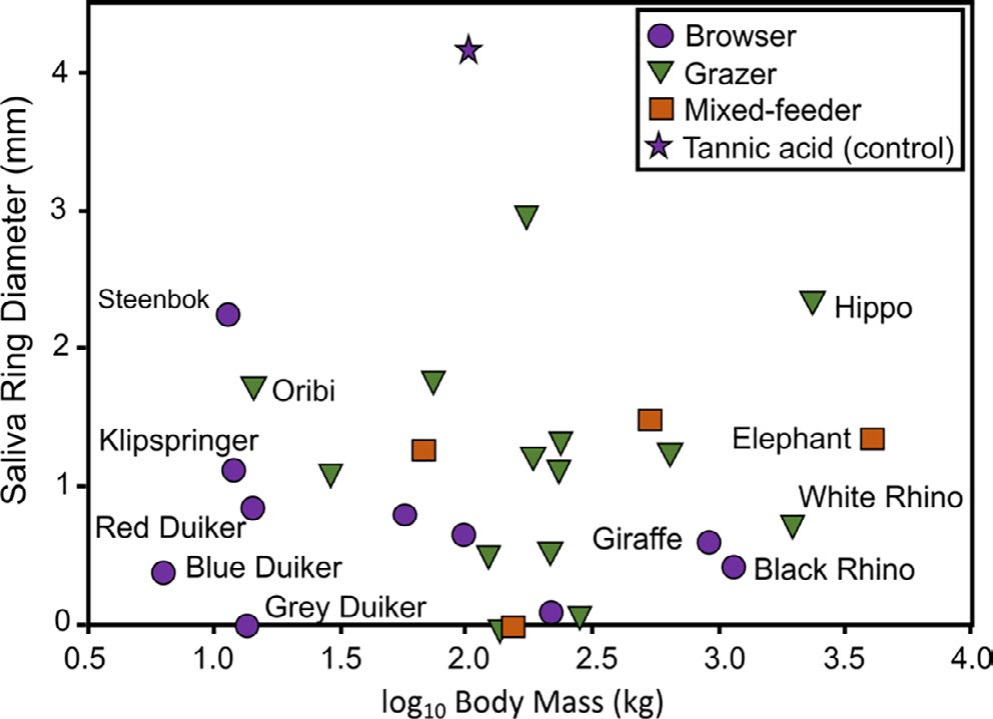
Mean saliva‐ring diameter of 28 African herbivorous mammals. All species bigger than 3 (>1,000 kg) are considered megaherbivores (listed). Small herbivores, most of which are browsers, are also listed. Purple star = tannic acid, the hydrolyzable tannin that acted as a control. Note that all species had saliva that contained proteins that reduced the diameter of the rings, indicating that they were capable of binding tannins. Some species could bind all tannins (mean value = 0 mm)

When we ignored the effect of phylogeny on the basis of a nonsignificant phylogenetic signal, we ran an ANCOVA and found that there was no significant difference between digestive system (ruminants vs. hindgut fermenters) (*F* = 1.139, *p* = .297) (Figure 4). There was also no significant effect of feeding niche (grazer vs. browser) (*F* = 0.007, *p* = .933), nor was there a significant interaction between digestive system and feeding niches (*F* = 1.910, *p* = .180). There was also no significant effect of the log_10_ body mass on mean saliva‐ring diameter (*r* ^2^ = 0.003, *F* = 0.082, *p* = .777) (Figure 5). There was also no significant relationship between mean saliva‐ring diameter and log_10_ body mass (*r* ^2^ = 0.004, *F* = 0.072, *p* = .791) for ruminants only.

All species could bind tannins, albeit to differing degrees (Table 1). Of those species that bound tannin completely (saliva‐ring diameter = 0 mm), one was a browser (gray duiker), one was a mixed feeder (bush pig *Potamochoerus larvatus*), and one was a grazer (red hartebeest *Alcelaphus busephalus*). Two species had a mean score close to 0 (=0.1), one of which was the greater kudu *Tragelaphus strepsiceros* (browser) and the other the roan *Hippotragus equinus* (grazer). The species least able to bind tannins was the gemsbok *Oryx gazella* (grazer; mean = 3.0 mm) compared to the tannic acid stock (control) solution mean of 4.18 mm (Figure 5). Of the small herbivores, five of the six species were browsers. The only exception was the oribi (grazer). Among the megaherbivores, the black rhinoceros and the giraffe *Giraffa camelopardalis* (both browsers) were most effective at binding tannins, but the saliva of the white rhinoceros (a grazer) was not that different at binding tannins from the two aforementioned megaherbivore browsers (Figure 5). Among the megaherbivores, the hippopotamus *H. amphibius* (strictly a grazer) was the least effective at binding tannins.

## 4 DISCUSSION

We found no evidence for proline‐rich proteins, although the inhibition radial diffusion assay that we used allowed us to establish that the saliva of all 28 herbivore species we tested are effective and can bind tannins (they reduced the size of the radial diffusion ring—Table 1). However, we were unable to establish the type/s of salivary proteins that they have. For the herbivores that we tested using the Beeley et al. (1991) technique, we can only say that those species do not have proline‐rich salivary proteins (Schmitt, 2017; Schmitt, Shuttleworth, Shrader, & Ward, 2020), but we do not know whether they are histatins or some as‐yet‐unidentified salivary protein (Lamy et al., 2008; Shimada, 2006).

Among mammals, salivary proline‐rich proteins that bind tannins occur in a number of orders, including the Artiodactyla, Rodentia, and Lagomorpha, as well as some herbivorous Marsupialia, but are absent among the Carnivora (Clausen et al., 1990; McArthur et al., 1991; Robbins et al., 1991). Even humans and other primates have tested positive for proline in their saliva (Bacon & Rhodes, 1998; Bennick & Connell, 1971; Mehansho, Butler, & Carlson, 1987), perhaps because of their historical dependence on tannin‐rich substances (e.g., berries, nuts, and many legumes (Foley & McArthur, 1994; Prinz & Lucas, 2000)). Similarly, domestic rats *Rattus norvegicus*, mice *Mus musculus,* and hamsters *Mesocricetus auratus* may also induce proline (Ann, Clements, Johnstone, & Carlson, 1987; Mehansho et al., 1983, 1987; Skopec, Hagerman, & Karasov, 2004). However, the domestic goat was not found to have proline compounds despite being a mixed feeder (eats both grass and woody plants; Austin et al., 1989; Distel & Provenza, 1991; Makkar, 2003; Schmitt, Ward, & Shrader, 2020; Ventura‐Cordero et al., 2017). Nonetheless, domestic goats are capable of binding tannins in their saliva, suggesting that another protein, as yet undescribed, is employed (Alonso‐Díaz, Torres‐Acosta, Sandoval‐Castro, & Hoste, 2010; Distel & Provenza, 1991; Lamy et al., 2008; Makkar, 2003; Schmitt, Ward, et al., 2020; Vaithiyanathan, Mishra, Sheikh, & Kumar, 2001; Ventura‐Cordero et al., 2017).

Among the Artiodactyla, several authors have found that browsing members of the Cervidae, such as mule deer *Odocoileus hemionus*, have proline in their saliva (Austin et al., 1989; Robbins et al., 1991). Among the Bovidae, the domestic cow *Bos taurus* is a grazing bovine and does have tannin‐binding proline proteins in its saliva (although this species is relatively ineffective in blocking tannins; Mole, Butler, et al., 1990). Yet, the closely related fellow bovines that we studied, including the browsers (greater kudu, nyala *Tragelaphus angasii*, bushbuck *T. scriptus*, and eland *Taurotragus oryx*) and grazing buffalos *Syncerus caffer* (Table 1), do not show evidence of proline. This might reflect a lack of sensitivity in the assay we used, although we think that this is unlikely to be the case. The pink/violet bands in the human saliva samples, highlighting the presence of proline‐rich proteins, were very clearly displayed on every gel, leading us to believe that the Beeley et al. (1991) technique was adequate for our study. Further research will be needed to establish whether these salivary tannin‐binding proteins could have evolved independently on 28 occasions, or whether a smaller number of evolutionary events led to their evolution.

We also predicted that there would be a significant effect of phylogeny on the ability of ruminants and nonruminants (hindgut fermenters) to block tannins. We found no support for this. Our phylogenetic analyses are consistent with earlier conclusions (Pérez‐Barbería, Gordon, & Illius, 2001; Robbins, Spalinger, & Van Hoven, 1995) that there was no support for the anatomical split between browsers and grazers on the basis of their gut morphologies. A continuum rather than a strict browser/grazer dichotomy may exist (Springer et al., 1997), which includes intermediate or mixed feeders between the two categories. The lack of divergence according to feeding niche (browser/grazer) may also apply to salivary proteins. An example that may be particularly pertinent is a study of salivary tannin‐binding proteins in three species of rhinoceros (Clauss et al., 2005). These authors found that the black rhinoceros (browser) had more effective salivary proteins for binding tannins than did the white rhinoceros (grazer) as one might expect. However, the Indian rhinoceros *Rhinoceros unicornis*, which is also a grazer, had more effective salivary proteins for binding tannins than either the black or white rhinoceros.

We predicted that browsers, which encounter tannins more frequently than grazers, would have a smaller saliva‐ring size, indicating a greater binding of tannins, resulting in greater reduction in their effectiveness (Austin et al., 1989; Hagerman et al., 1992; Robbins, Hanley, et al., 1987; Robbins, Mole, et al., 1987; Schmitt et al., 2016; Windels & Hewitt, 2011; Figure 3). However, we did not find a significant difference between these two groups. We compared nonruminants (hindgut fermenters) and ruminants and found no difference in the tannin‐binding ability that has been suggested to be driven by these anatomical differences between browsers and grazers (Hofmann, 1973, 1989; Hofmann & Stewart, 1972). We also found no correlation between the relative effectiveness of tannin‐binding proteins in the saliva and body mass. This result is inconsistent with data, indicating that there was scaling of salivary gland size and body mass^0.75^ and thus an assumed greater production of salivary tannin‐binding proteins by larger herbivores (Hofmann et al., 2008). Our results are also inconsistent with those of Hofmann (1973) and Kay, Engelhardt, and White (1980) who found that grazing ruminants had parotid salivary glands that were three times smaller (and thus likely produced less salivary tannin‐binding proteins) than those of browsing ruminants that constantly feed on tannin‐rich food. Yet, there is conflicting evidence, in any case, on the effectiveness of proline contained in binding tannins (Mole, Butler, et al., 1990), which means that scaling of salivary gland size to salivary tannin‐binding protein may not be that meaningful. For example, the domestic cow (a grazer) has high amounts of proline in its salivary glands, yet does not bind tannins any more effectively than another common grazer, the domestic sheep, that has low amounts of proline in its salivary glands (Mole, Butler, et al., 1990). These authors suggested that proline‐rich salivary proteins may not have evolved solely for the purpose of binding tannins but may also be present in the saliva to stabilize minerals in the teeth or to limit tooth decay (Bennick, 1982). However, that does not explain why many herbivores possess proline and carnivores do not (Austin et al., 1989; Harborne, 1993; Hofmann et al., 2008; Robbins et al., 1991).

Of the small‐bodied species we studied, the majority are browsers (red duiker, gray duiker, blue duiker, steenbok, and klipspringer) and often encounter foods that are very tannin‐rich. The only exception among the small antelopes was the oribi, a grazer (Arcese, Jongejan, & Sinclair, 1995; Everett, Perrin, & Rowe‐Rowe, 1992; Stears & Shrader, 2020). These small herbivores are closely related (Figure 2), suggesting some phylogenetic effect. However, the relative abilities of these small herbivores to bind tannins were not necessarily greater than those of several (larger) grazers (Figure 5), substantiating the absence of evidence for phylogenetic effects. Furthermore, the paradox that the oribi is the only small antelope of six species that is a grazer (i.e., eating low‐quality food) is worth re‐examining. We speculate that the oribi is at the body size threshold for being a grazing ruminant (Clauss et al., 2003; Demment & Van Soest, 1985; Gordon & Illius, 1994). Future studies should determine whether the effectiveness of the salivary proteins for binding tannins is integral to the fact that five of the six small herbivores are browsers, or whether behavioral avoidance by virtue of their small mouthparts (Iason & Villalba, 2006; Nobler et al., 2019; Provenza & Balph, 1987) can explain their abilities to access high‐value food items.

Although small browsers might be expected to have evolved salivary tannin‐binding proteins because of their high metabolic rates, we have shown elsewhere (Schmitt et al., 2016) that even animals as large as elephants have saliva that can bind tannins to maintain their large body masses (see also Dierenfeld, Du Toit, & Braselton, 1995; Furstenburg & Van Hoven, 1994; Muller, 2013; Owen‐Smith & Chafota, 2012; Schmitt et al., 2018; Shaw, 2011; Ward, Muller, & Shrader, 2017). All of the five African megaherbivores that we studied have protein(s) in their saliva that bind to tannins, regardless of whether they are browsers (giraffe and black rhinoceros), mixed feeders (elephants), or grazers (hippopotamus and white rhinoceros; Figure 5). Only two species of megaherbivores are closely related (black and white rhinoceros; Figure 2). Thus, there is no phylogenetic effect. This suggests that the relationships between body size and the quality of food (as determined by plant secondary metabolites) required by large herbivores should be re‐examined.

## CONFLICT OF INTEREST

The authors declare no competing interests.

## AUTHOR CONTRIBUTION

**David Ward:** Conceptualization (equal); Data curation (lead); Formal analysis (lead); Funding acquisition (lead); Investigation (equal); Methodology (equal); Project administration (equal); Writing‐original draft (lead); Writing‐review & editing (lead). **Melissa Schmitt:** Conceptualization (equal); Data curation (equal); Methodology (equal); Resources (equal); Validation (equal); Visualization (equal); Writing‐original draft (supporting); Writing‐review & editing (supporting). **Adrian Shrader:** Conceptualization (equal); Investigation (equal); Project administration (equal); Resources (equal); Supervision (equal); Validation (equal); Visualization (equal); Writing‐original draft (supporting); Writing‐review & editing (supporting).

## Data Availability

All information is present here (Table 1; Figure 2).
